# Minutes of the Ohio State Medical Society, Held in the City of Columbus, June, 1851, with Addresses and Essays

**Published:** 1852-02

**Authors:** 


Art. V.—*Minutes of the Ohio State Medical Society*, held in the city of Columbus, June, 1851, with Addresses and Essays. Columbus, 1851. 8vo pp. 152.

The Minutes of the Society are brief; the reports, addresses, and essays make one hundred and fifty pages of the volume, and among them are several of decided ability and interest.

The first report is on the Medical Societies of Ohio, and the committee close it with the following resolution:

"*Resolved*, That the encroachments made upon the medical profession, by quackery, in its various forms, have greatly lessened the standing and usefulness of the regular profession of medicine, in the public confidence—that this is greatly owing to our holding in our own connection, men who are known to be deficient in medical attainments and qualifications—that a thorough organization, under proper and wholesome restriction, in the form of Medical Association, is the most efficient means of preventing and counteracting this evil."

The first essay is by Dr. Bœrstler, on the the contagiousness of cholera, of which fact he entertains no doubt. He cites many facts favorable to his conclusion; but he cannot be ignorant that just as many might be cited which look in precisely an opposite direction.

The report of the committee on *ovarian diseases* embraces interesting statistics, and concludes with the following summary:

"1. That even extensive sections of the peritoneum, are not so often followed by fatal results, nor are they so hazardous, as was formerly apprehended by surgeons.

2. That a greater number of permanent cures have resulted from extirpation, than from any other mode of treatment.

3. That the encysted variety of ovarian tumors, is most favorable for operation."

Appended to the report is a communication from Prof. Atlee, giving an analysis of two hundred and twenty-two cases of ovariotomy, which will be examined with great interest by the surgeon. And this is followed by a report of five cases of ovarian tumors, by Dr. Buckmaster.

The next paper is by Dr. Buckner, and gives the particulars of an anomalous case of abdominal tumor. To this, various reports succeed, of one of which, "on the use of opium in certain forms of nervous irritibility," &c., we avail ourselves in another department of our present number.

A very interesting essay on Typhoid Fever, by Dr. John Dawson, follows, from which, if we had room, we would be glad to make copious extracts. Dr. Dawson is well known to our readers, having contributed many valuable papers to our journal. The present essay is carefully written, and evinces at once a turn for patient observation and a talent for philosophising of the utmost value in the practitioner of medicine. In respect to style, we should say that it is an improvement upon some of his earlier papers. The style is less involved; more simple, and unaffected; and therefore, to our tastes, more agreeable. We have remarked only a few such sentences as the following in this essay:

"Invoked, if possible, with still more confidence, have been the alterations of structure, which are alleged to be peculiar to typhoid fever."

With an occasional sentence like this, we find the words "therapeia" and "dietetica," for therapeutics and dietetics, to which, if we felt disposed to be critical, we might object a little; but where an essay possesses so many excellent qualities, the critic may well pass over a few trifling blemishes like these in silence.

Reports on "Practical Medicine" and the "Medical Literature of Ohio," complete the volume. A well merited compliment is paid in the latter to Dr. Drake's work, on the "Principal Diseases" &c., of which the committee say, that, "until the face of the vast regions covered by his work shall have experienced a physical change, this author will be referred to by all writers upon general medicine, in the United States."

